# Recontact in clinical practice: a survey of clinical genetics services in the United Kingdom

**DOI:** 10.1038/gim.2015.194

**Published:** 2016-02-18

**Authors:** Daniele Carrieri, Anneke M. Lucassen, Angus J. Clarke, Sandi Dheensa, Shane Doheny, Peter D. Turnpenny, Susan E. Kelly

**Affiliations:** 1 Egenis, University of Exeter, Exeter, UK; 2 Faculty of Medicine, University of Southampton, Southampton, UK; 3 School of Medicine, University of Cardiff, Cardiff, UK; 4 Royal, Devon, and Exeter Hospital, Exeter, UK

**Keywords:** clinical genetics, ethics, next-generation sequencing, recontact, survey

## Abstract

**Purpose:**

To ascertain whether and how recontacting occurs in the United Kingdom.

*Genet Med* **18** 9, 876–881.

**Method:**

A Web-based survey was administered online between October 2014 and July 2015. A link to the survey was circulated via an e-mail invitation to the clinical leads of the United Kingdom's 23 clinical genetics services, with follow-up with senior clinical genetics staff.

*Genet Med* **18** 9, 876–881.

**Results:**

The majority of UK services reported that they recontact patients and their family members. However, recontacting generally occurs in an *ad hoc* fashion when an unplanned event causes clinicians to review a file (a “trigger”). There are no standardized recontacting practices in the United Kingdom. More than half of the services were unsure whether formalized recontacting systems should be implemented. Some suggested greater patient involvement in the process of recontacting.

*Genet Med* **18** 9, 876–881.

**Conclusion:**

This research suggests that a thorough evaluation of the efficacy and sustainability of potential recontacting systems within the National Health Service would be necessary before deciding whether and how to implement such a service or to create guidelines on best-practice models.

*Genet Med* **18** 9, 876–881.

## Introduction

### The problem

The increasing introduction of new genetic technologies in the investigation of patients is creating much new information. This raises important issues related to the communication of the potential health significance of new findings (e.g., new information about the natural history of a condition; surveillance or treatments available; improved diagnostic accuracy, such as a new test; or new information about previously uncertain test results, such as classification of a variant of unknown significance). This might mean that patients examined and tested in the past could now be offered more informative testing. As a result, questions may arise about whether health-care professionals, such as clinical genetics specialists, have a responsibility or duty to recontact former patients. Innovations in genomic medicine can have significant implications for patients and families regarding health, reproductive decisions, lifestyle choices, employment, and psychosocial well-being. However, recontacting patients may also affect them negatively, potentially causing anxiety and concerns over health and economic activity, and they may consider it an intrusion of privacy.^1^ Recontacting patients has been raised as a major issue in medical genetics, but its importance will become even more widespread with the increasing integration of genomics in medicine.^2^ Clarifying the issues of whether and how recontacting in clinical genetics should be implemented is of importance to the current information revolution in health care.^3^

### Policy/guidelines

There is no professional consensus in clinical genetics about whether, or how, former patients should be recontacted when new genetic information relevant to them or their family members arises from the use of new technologies. A survey of regulations and practices of genetic counseling in 38 European countries found that recontacting was among the least covered topics in both national legislation and applied practice guidelines.^4^ The only guideline currently available is a statement originally published in 1999 by the American College of Medical Genetics.^5^ This document highlights the logistical difficulties of locating and recontacting former patients, and it identifies the primary-care provider—the specialist with the task of providing continuing care, such as the general practitioner (GP) in the UK National Health Service (NHS)—as the principal responsible health-care provider to alert patients to the need for recontact if necessary. Genetics service providers would be responsible for providing clinical updates to patients in rare cases in which they are offering continuing care. The statement also suggests that patients be appropriately advised to update their primary-care provider or the genetics service provider if relevant changes in their lives occur, such as pregnancy.^5^ The 2008 revision of the statement recognized that, with the uptake of next-generation sequencing, testing laboratories may now be in a position to know about changes in interpretation of variants whose significance had previously been unknown (former variant of unknown significance) or about reclassifications of previously classified variants, and they should make an effort to contact relevant health-care providers if new information changes the previous clinical interpretation of a sequence variant.^6^

### Systematic review of recontacting literature

The only systematic review of recontacting literature^7^ identified 61 articles published between 1991 and 2014 that explored ethical, legal, social, psychological, clinical, and practical issues. The review identified a divergence of expectations about who is responsible for keeping patients up to date with relevant information. While patients tend to assign this responsibility solely to health-care professionals—especially genetics service providers—health-care professionals tend to think this responsibility should be shared by patients.^8,9,10^ The review also showed that, although most patients want to be recontacted, not all do, because of the potential for strong emotional reactions to such recontact.^11,12^

Most authors identified a clash between the ethical desirability and the practical difficulty of recontacting. The most common practical barriers to recontacting mentioned are lack of infrastructure for tracking data for former patients (e.g., digitalization of databases);^13^ lack of time and resources (e.g., staff, money) to perform recontacting;^8,9,14^ and not having patients' current addresses.^15^

Suggestions for overcoming these barriers include implementing digital communication systems between laboratory, clinicians, and patients;^14^ involving patients in the processes of recontacting;^10,16^ and involving patient support or advocacy groups.^8,17^

### Need for more empirical evidence

The authors of the systematic review suggest that more empirical evidence is needed to advance the discussion about whether and how recontacting should be implemented.^7^ There is limited empirical evidence concerning the perspectives of health-care professionals and patients on recontacting or what is occurring in clinical practice. There is an urgent need for more research on the practical and ethical implications for health-care systems regarding recontacting recommendations or guidelines. To begin to provide such empirical evidence, we conducted a survey of clinical genetic services in the United Kingdom regarding their current recontacting practices.

## Materials and Methods

### The survey

The survey we conducted is part of an ongoing study that investigates: the place of recontacting in current clinical practice in the NHS in the United Kingdom; the ethical, legal, and social issues raised; and the expectations of patients and health-care professionals concerning recontacting (study website: http://ex.ac.uk/mgc). The main objective of the survey reported here was to ascertain whether and how recontacting occurs in the United Kingdom. The topic areas were identified from debates in the relevant literature. The questions were developed from relevant literature, the clinical experience of members of the research team, and a pilot survey on recontacting conducted by some members of the team in 2011 as part of the “Development of a Draft NHS Information Standard for Genetics” by the National Genetics Reference Laboratory in Manchester, England (http://www.ngrl.org.uk/Manchester/newsitem/project-develop-draft-nhs-information-standard-genetics-launched). The questions were further refined by pilot testing the survey with clinical genetics service providers.

The Web-based survey, designed using the Bristol Online Survey tool, was administered between October 2014 and July 2015. A link to the survey was circulated via an e-mail invitation to the clinical leads of the United Kingdom's 23 clinical genetics services, with a follow-up e-mail sent to senior clinical genetics staff. The landing page of the survey site contained information about the study and the research team, and it invited respondents to discuss the survey with colleagues in their service before completing it.

The survey included closed and open questions with expandable text boxes to elicit explanatory comments, and examples. This combination of closed and open questions allowed the research team to collect both quantitative and qualitative data. Free-text responses from the survey were analyzed using thematic analysis.^18^ The survey is provided in the **Supplementary Information** online.

The study was approved by the University of Exeter's Social Sciences and International Studies Ethics Committee.

## Results

### Respondent characteristics

Twenty of the 23 clinical genetics services in the United Kingdom (for a complete list of clinical genetics services, including laboratories, in the United Kingdom visit: http://www.bsgm.org.uk/information-education/genetics-centres/) completed the survey, from all four nations comprising the United Kingdom. Of the 20 respondents who completed the survey (one per service), nine were consultant geneticists, seven were consultant genetic counselors, and the remaining four were genetic counselors.

### Recontacting experiences and practices

The majority of the services (19/20) reported recontacting patients and relevant family members because of significant new information. However, 16 of 20 services indicated that recontacting occurred on an occasional basis. Only three services reported that they routinely recontact former patients.

A variety of reasons were given for having recontacted patients (see **Table 1**). The most common were availability of new tests or new results; new clinical guidance; and reclassification of a variant of unknown significance. **Table 1** reports the main themes from respondents' answers, with some quotations to illustrate each theme.

Only seven services responded that they have developed recontacting procedures; three of these were the centers that responded that they routinely recontact former patients. The procedures reported varied across these centers; there was no significant pattern. Common across these responses was the mention of a lack of a codified procedure for recontacting. Two centers reported that they inform a patient's GP or pediatrician as appropriate; the two others reported that they inform the patient directly (via letter or telephone call). The three remaining centers mentioned that they use clinical databases that are held by NHS services (when undergoing testing, patients consent to being in the database and indicate whether they wish to be recontacted). However, one of these three centers added that they were experiencing problems with their clinical databases—as a result of having moved to electronic notes—and that they were using external databases as well.

### “Triggers” for recontacting

We asked about the type of information that would be sufficiently relevant to trigger recontacting (giving the example of relevance based on clinical actionability of the information or on its analytic validity). The majority of the services (14/20) indicated that one of the most important elements would be clinical actionability, i.e., information that has an impact on the clinical management of the patient and the patient's family. One service added that in practice they would recontact only if new information relates to a small group or an individual patient, because of the workload of recontacting large numbers of patients. Other common answers included the publication of new or revised guidelines or new laboratory reports.

### Use of clinical databases

The majority of the responding centers (18/20) use existing clinical databases rather than bespoke recontacting systems for recontacting purposes. Of these, nine answered that they use the databases mainly to identify patients; the other nine used them to review notes and/or to flag patients for recontacting. The clinical database respondents referred to are databases held by the UK NHS. They are used mainly for clinical purposes, but sometimes they are used to identify patients for research. Their current format is mixed (some centers use electronic versions; others use paper-based versions).

### Recording of patient preferences

The majority of services (12/20) indicated they do not routinely ask patients about their recontacting preferences as part of the procedure for obtaining informed consent for genetic testing. Of these, six services responded that they record patients' recontacting preferences systematically, six responded that they do so only occasionally, and the remaining eight responded that they do not record patients' preferences at all. Consent forms for genetic testing vary across the services in the United Kingdom. Six services said they used consent forms that give the option to patients to express their recontacting preferences. However, as one of these respondents added, consent forms are not always used in clinical practice (**Table 2**).

Seven services stated that the main reasons for not recording patients' preferences—or for not mentioning the possibility of recontact to patients at all—were lack of resources available to offer a recontacting service and concern about raising unrealistic expectations in patients. Four services mentioned their “open-door policy,” whereby clinicians encourage the patient to get back in touch with the genetics department from time to time to check if there are advances or to update clinicians about important changes in the family.

When asked hypothetically if there were reasons to recontact patients even when patients had indicated they would not want to be recontacted, a majority of services (14/20) responded that they would. In line with previous answers about the type of information that would trigger recontacting, the main reason provided was new information that may have clinical implications for patients and family members. The GP was explicitly mentioned by four respondents as a (first) point of contact to involve in this process. One respondent argued that patients' decisions not to be recontacted could not necessarily anticipate the specific information subsequently available and therefore might be an insufficiently informed refusal.

Of the six services stating that they would respect patients' preference not to be recontacted, one added that if new information was likely to have significant implications for a family member, then they would inform the patient's GP; two mentioned the possibility of seeking external advice, e.g., from the Medical Defence Union or the Genethics Club (http://www.ndph.ox.ac.uk/research/ethox-centre/research-projects/clinical-ethics/the-genehthics-club), a national forum in which health professionals discuss practical ethical problems encountered in the working lives of clinical genetics departments in the United Kingdom. One respondent made the point that, although patients should not be recontacted if they clearly expressed this preference, in practice very few patients would be definite about their preferences.

### Implementation of recontacting systems

A slight majority of services (11/20) were unsure about whether routine recontacting systems should be implemented. Of the remaining services, five indicated that they should be implemented and four said that they should not be.

The main arguments in favor of implementing systems for recontacting revolved around the ideas that this would improve the quality of care received by patients, it would increase patients' autonomy, and it might reduce the potential for litigation. We report here some illustrative quotations.

“New information may help reduce the risk of disease/mortality to other family members—e.g. where a mutation is identified—screening and treatment may be offered to those at risk.”

“Technology and knowledge are increasing rapidly and what was known or possible even a short time ago may well be different and allow individuals more choice or better risk assessment or treatment.”

One respondent also noted that clinical genetics would be the only medical specialty in a position to offer recontacting. Another respondent highlighted that having recontacting mechanisms in place would facilitate the process.

Among the arguments against the implementation of recontacting systems, lack of resources was again mentioned and linked to equitable provision of services within the NHS. Another common argument was that patients should be encouraged to be more responsible for their health and share the responsibility with clinicians to keep current with relevant medical information.

Legal implications were also mentioned as an argument against the implementation of recontacting systems. Specifically, the concern was that introducing such systems would create a standard practice and that failure to recontact could then be seen as negligent.

Concern was expressed by one service that recontacting might cause anxiety for patients and family members; another service mentioned that recontacting would be more difficult to implement because genetic testing is increasingly ordered by mainstream medical specialties. Finally, one service said that the time frame for recontacting responsibilities would need clarification; for example, for how many years from the genetic test would such a responsibility apply?

## Discussion

This was the first study in the United Kingdom to explore current recontacting practices in clinical genetic services.

### Recontacting occurs, but on an ad hoc basis

The majority of UK services reported that they recontact patients and family members, confirming the significance of recontacting for good quality of care in clinical practice. The majority of respondents indicated that “clinical actionability” of the new information is the main reason to recontact. Respondents' answers also suggest that recontacting is becoming more important as clinical whole-genome approaches deliver many more genetic variants for interpretation. Recontacting tends to occur in an ad hoc fashion when an event triggers clinicians to review a file, rather than systematically as part of routine clinical practice.

### Procedures vary greatly across the United Kingdom

Our findings suggest that there are no standardized recontacting practices operating in the United Kingdom. This diversity may be the result of historical and resources allocation differences. Overall, the fact that recontacting does occur but not in a standardized fashion reflects the tension identified in prior research between the desirability of recontacting and the current lack of mechanisms and resources to offer it more systematically, or at all, resulting in unequal recontacting service provision across the country.^8,19^ Only a few centers that recontact patients have developed systematic recontacting procedures, but these procedures vary.

We also found considerable diversity in practices regarding the use of clinical genetic databases for recontacting purposes (e.g., to retrieve patients' contact details, to review notes, to flag patients for recontacting) and regarding how and whether patients' recontacting preferences are recorded.

### Patients' preferences and professionals' responsibility

Our findings suggest tension between respecting patients' preferences to not be recontacted (i.e., their right not to know) and the responsibility or duty of care of health-care professionals toward patients and family members. Although very few clinical genetics services reported that they record patients' recontacting preferences systematically, in response to a hypothetical question many services responded that there were circumstances in which they would recontact patients and family members even if the patients had indicated they would not want them to do so. These circumstances were related to the emergence of new, clinically actionable information that could have an impact on the health management of the patient or family members. Respondents' views appear to be in line with the European Society of Human Genetics recommendations on the use of whole-genome sequencing in health care,^20^ particularly with the recommendation that “Patients' claims to a right not to know do not automatically over-ride professional responsibilities when the patient's own health or that of his or her close relatives are at stake” (p. 583).

### Implementation of recontacting

Finally, although the majority of respondents indicated that recontacting occurs in their genetic centers, albeit in a nonsystematic way, more than half were unsure about whether recontacting systems should be implemented. This finding suggests that a thorough evaluation of the desirability, efficacy, equitability, and sustainability of potential recontacting systems in the NHS would be necessary before deciding whether and how to implement such a service, or before suggesting guidelines. Agnosticism toward the implementation of recontacting systems was accompanied, for some, by the suggestion of greater patient involvement in the process of recontacting. This idea was supported as promoting patient autonomy while circumventing resource and infrastructural barriers that may prevent health-care professionals from offering efficient recontacting services. Some framed this idea as being less paternalistic than a clinician-driven implementation model in which the responsibility for keeping patients updated is placed solely on health-care professionals. However, it is important to point out that a clinician-driven implementation model would not be paternalistic if patients choose it. Further, shifting responsibility for recontacting from clinicians to patients may be seen as promoting patient choice but may not necessarily eliminate a potential duty clinicians could have to recontact patients. (We follow the definition of “duty to recontact” given in the first systematic review of this issue.^7^ Such a duty is defined as the ethical and/or legal obligation to recontact former patients in light of new genetic information.)

Our data provide some insight into genetics service providers' worries about legal consequences of implementing recontacting systems. Specifically, some respondents expressed the concern that introducing recontacting systems would create a practice standard and that failure to recontact could then be seen as negligent.

It is important to point out that—although in the United Kingdom there is currently no statute law and professional guidance regarding recontacting, and although there have been no known litigation cases—the fact that some centers do recontact might be seen as creating a duty to do so. If recontacting becomes standard practice, then patients who are not recontacted, and are thus not able to avail themselves of interventions that might benefit them, could claim that a reasonable health-care professional should provide this service and that the reasonable patient could expect it. Moreover, although there is no statute law or cases about recontact in genetics, there have been cases in North America relevant to recontact in other areas of medicine.^16,17^ It is difficult to determine whether concern about potential medicolegal consequences influences health-care professionals' practices. We are exploring these issues in interviews that we are currently conducting with health-care professionals potentially involved in recontacting (clinical geneticists and other mainstream specialties) in the United Kingdom.

### Limitations

There are some limitations of this study. Although we asked respondents to discuss the questions with their center's clinical team before completing the survey, we cannot know whether they did. As genomics enters mainstream practice, it will be important to seek wider representations of professional views and experiences. To address these limitations, we are conducting further research to investigate the views of health-care professionals potentially involved in recontacting (including specialties other than clinical genetics), patients, and other stakeholders, such as patient-support groups.

This survey was administered in the United Kingdom and reports the views and concerns of genetics service providers working in this country. The medicolegal aspects of recontacting and health-care professionals' views on the issue are likely to be subtly different in other countries, especially where legal systems are different (e.g., Roman law as opposed to common law–based systems). It will be important to conduct a wider analysis to support recommendations or practice guidelines, if any, in this increasingly complex area of clinical practice.

## Disclosure

The authors declare no conflict of interest.

## Figures and Tables

**Table 1:**
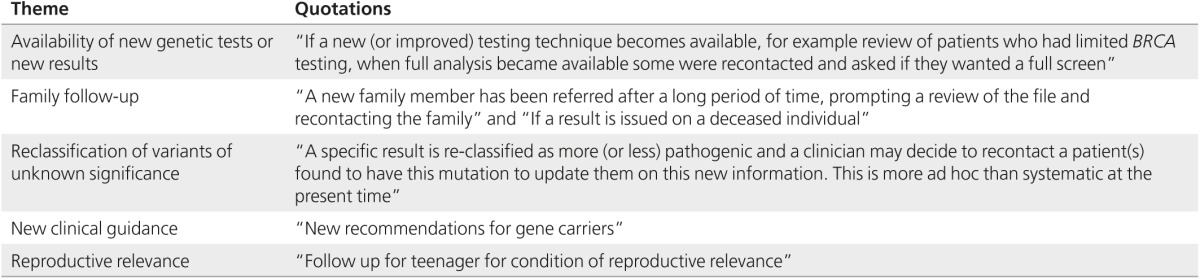
Most common reasons for recontacting patients and relevant family members (grouped by themes)

**Table 2:**
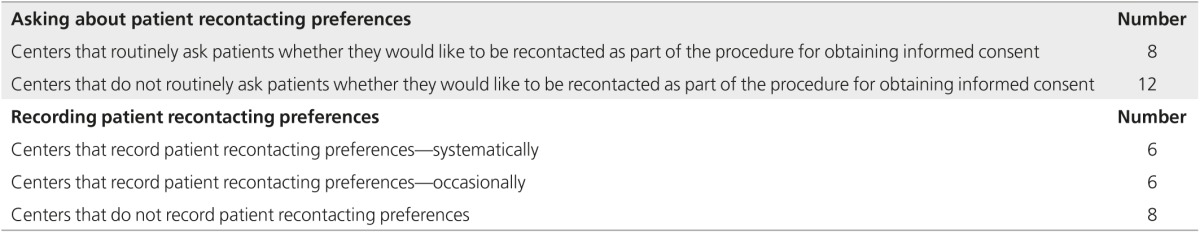
Number of centers that ask and record patient recontacting preferences
