# Supplementary material for: Recontact in clinical practice: a survey of clinical genetics services in the United Kingdom
Source: Genet Med. 2016 Feb 18;18(9):876–81. doi: 10.1038/gim.2015.194 (PMC5052431; doi:10.1038/gim.2015.194)
Supplement: Supplementary Information [file gim2015194x1.pdf]

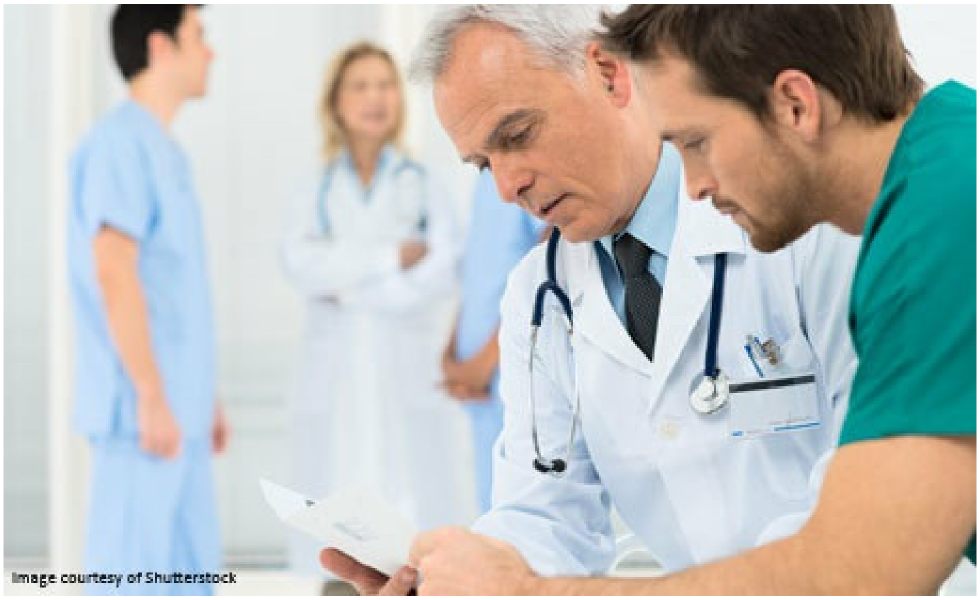

MAINSTREAMING GENOMICS: RE-CONTACTING PATIENTS IN A DYNAMIC HEALTHCARE ENVIRONMENT

INFORMATION FORM FOR SURVEY OF GENETICS SERVICES

Title of Research Project
Mainstreaming Genomics: Re-contacting patients in a dynamic healthcare environment

Details of Project
This project aims to investigate whether, and in what ways, there might be a duty, or responsibility, to re-contact patients when new information about their genetic status or health condition(s) comes to light. In the initial phase of this project we are collecting information from UK genetic services about existing infrastructure and practices relevant to re-contacting patients. This information will form the empirical background to the remainder of the project. The project is

an interdisciplinary effort between medical sociologists, an academic lawyer, and clinical genetic professionals. The results of the project will contribute to ethical, legal, clinical and policy debates concerning the integration of genetic and genomic information across the health service. Data from this survey will inform the overall findings and recommendations disseminated to practice and policy communities.

Project Funding

The project is funded by the Economic and Social Research Council (ESRC). The project commenced on May 1, 2014, and will run for 3 years.

Contact Details

For further information about the research or your interview data, please contact:

Dr Susan E. Kelly, Department of Sociology, Philosophy & Anthropology, Exeter University, UKTel 00 44 (0) 1392 725139, S.E.Kelly@ex.ac.uk

If you have concerns/questions about the research you would like to discuss with someone else at the University, please contact:

Professor David Inglis, Director of Research for the Department of Sociology, Philosophy & AnthropologyTel 00 44 (0) 1392 725682, D.Inglis@exeter.ac.uk

Taking Part

Taking part will consist of completing a brief survey composed of 12 simple questions. No personal information will be requested, recorded or maintained.

Confidentiality

Survey data collected will be held in confidence. They will not be used other than for the purposes described above and third parties will not be allowed access to them (except as may be required by the law). No personal data will be collected -- only service unit level data will be collected. We are happy to report summary data to participating units that request this information. All data will be held in accordance with the Data Protection Act.

Anonymity

Survey output will not mention individual names or personal information.

MAINSTREAMING GENOMICS: RE-CONTACTING PATIENTS IN A DYNAMIC HEALTHCARE ENVIRONMENT

The Issue

With the rapid technological advances in genetics and genomics, new relevant information is becoming increasingly available (e.g. new information about natural history of a condition, available surveillance or improved diagnostic accuracy or new information about previously uncertain test results). Healthcare professionals will sometimes become aware that patients they have seen in the past might now benefit from more up to date information and may want to re-contact them. By "re-contacting" we mean contacting patients who have previously been discharged from a genetic service.Clarifying the issue of re-contacting is of importance to the current information revolution in healthcare. Academic literature and clinical statements report a tension between the desirability - fromprofessional and ethical points of view - and the logistical practicality of re-contacting patients and families. Logistical issues are also compounded by considerations of privacy, confidentiality and responsibility.

The Survey

We are interested in your current approaches to these situations. The survey has YES and NO questions and open text boxes for you to provide more information. The text boxes will expand to fit your answers. We would like you to complete the survey on behalf of your Clinical Genetic Service. Therefore, we suggest you discuss this survey with your colleagues in your service team before filling it.

Thank you very much.

Questions are **mandatory** unless marked otherwise.

Note that once you have clicked on the CONTINUE button your answers are submitted and you cannot return to review or amend that page.

MAINSTREAMING GENOMICS: RE-CONTACTING PATIENTS IN A DYNAMIC HEALTHCARE ENVIRONMENT

1 Your Clinical Genetic Service

2 Your professional role (e.g. consultant, training grade, genetic counsellor, etc.)

3 The country you work in

4 Date you are completing this survey

Dates need to be in the format 'DD/MM/YYYY', for example 27/03/1980.

Please make sure the date is between 04/08/2014 and 01/12/2015.

(dd/mm/yyyy)

5 Are patients (and relevant family members) re-contacted in your clinical genetic service when new relevant information becomes available?

- ○ YES - routinely
- ○ YES - occasionally
- ○ NO

5.a If YES, what are the most common reasons for re-contacting patients (and relevant family members)?

5.b If NO, why not?

5.c If YES, has your clinical genetic service developed procedures for re-contacting?

- ○ YES
- ○ NO

5.c.i If YES, please describe the procedures

6 What type of information does your clinical genetic service consider to be relevant enough to trigger a re-contact? (e.g. is relevance based on the clinical actionability of the information, or on its analytic validity?)

7 Do you use your clinical databases for re-contacting purposes?

- YES
- NO

7.a If YES, how do you use the databases (e.g. to access patient address only, as a mechanism to review notes/flag patients etc.)

8 Do healthcare professionals in your clinical genetic service routinely ask patients whether they would like to be re-contacted as part of the procedure for obtaining informed consent?

- ○ YES
- ○ NO

9 Do you record patient wishes about re-contacting? (e.g. do not re-contact; re-contact if advances in clinical genetics can or might help, etc.)

- ○ YES - systematically
- ○ YES - occasionally
- ○ NO

9.a Could you elaborate on why/how this is/is not part of your current practice?

10 If patients indicate they do not want to be re-contacted (or they do not want their relevant family members to be re-contacted), do you consider there might be reasons healthcare professionals should re-contact them anyway?

- ○ YES
- ○ NO

10.a If YES, what are these reasons?

10.b If NO, why not?

11 Do you think clinical genetic services should implement routine re-contacting systems?

- YES
- NOT SURE
- NO

11.a What do you think would be the main arguments for?

11.b What do you think would be the main arguments against?

12 Please add any further comments and observations

Final Page

Thank you for completing the survey. If you have any questions, please email: recontact-genomics@exeter.ac.uk

For more information on the project, please visit: http://ex.ac.uk/mgc

---
